# Panax notoginseng preparation plus aspirin *versus* aspirin alone on platelet aggregation and coagulation in patients with coronary heart disease or ischemic stroke: A meta-analysis of randomized controlled trials

**DOI:** 10.3389/fphar.2022.1015048

**Published:** 2022-12-07

**Authors:** Lulu Dai, Ying Zhang, Yuerong Jiang, Keji Chen

**Affiliations:** National Clinical Research Center for Chinese Medicine Cardiology, Xiyuan Hospital, China Academy of Chinese Medical Sciences, Beijing, China

**Keywords:** panax notoginseng preparation, aspirin, panax notoginseng saponins, panaxatriol saponin, platelet aggregation, coagulation, meta-analysis

## Abstract

**Purpose:** We aimed to evaluate the effects of Panax notoginseng preparations (PNP) containing Panax Notoginseng Saponins (PNS) or Panaxatriol Saponin (PTS) on platelet aggregation and coagulation in the adjuvant treatment of coronary heart disease (CHD) and ischemic stroke (IS).

**Methods:** Randomized controlled trials (RCTs) comparing the combination of PNP and aspirin (ASA) *versus* ASA alone for CHD or IS were searched in eight databases. Subgroup analysis was performed according to saponin category. When statistical heterogeneity was significant, sensitivity analysis was performed using the leave-one-out approach. Funnel plot, Egger’ test, and Begg’ test was adopted to detect publication bias.

**Results:** Twenty RCTs involving 2216 patients were analyzed. Compared with ASA alone, PNP plus ASA had a stronger inhibitory effect on in PAgR [PNS, WMD = −6.10 (−7.25, −4.95), *p* < 0.00001; PTS, WMD = −3.53 (−4.68, −2.38), *p* < 0.00001]; PNS plus ASA better reduced FIB [WMD = −0.43 (−0.49, −0.36)] and DD [WMD = −0.59 (−0.67, −0.51), *p* < 0.00001], while PLT (*p* = 0.07) and PT (*p* = 0.34) were not significantly different; PTS plus ASA better prolonged PT [WMD = 1.90 (1.47, 2.32), *p* < 0.00001] and PT-INR [WMD = 0.22 (0.11, 0.32), *p* < 0.0001], whereas no significant difference in DD (*p* = 0.1) and bleeding-related events (positive fecal occult blood, *p* = 0.96; upper gastrointestinal bleeding, *p* = 0.67; subcutaneous hemorrhage, *p* = 0.51; bulbar conjunctival hemorrhage, *p* = 0.51; hematuria, *p* = 0.58). There was no significant difference between PNP plus ASA and ASA alone in terms of gastrointestinal side effect (PNS, *p* = 0.65; PTS, *p* = 0.56) and urticaria (PNS, *p* = 0.57; PTS, *p* = 0.55).

**Conclusion:** PNP combined with ASA might produce stronger antiplatelet aggregation and anticoagulation effects without increasing bleeding risk, gastrointestinal side effects, and urticaria compared with ASA alone.

**Systematic Review Registration:** https://www.crd.york.ac.uk/PROSPERO/#recordDetails, identifier CRD42022339234.

## 1 Introduction

Panax notoginseng (Sanqi), the root of Panax notoginseng (Burk.) F. H. Chen, is a traditional Chinese medicine with a long history of medicinal use in China. Panax notoginseng contains more than 200 metabolites, including saponins, polysaccharides, dencichine, flavonoids, and fatty acids (Wang M M et al., 2016), which can be mainly classified into two categories: saponins and non-saponins, and saponins are not only the main metabolites of Panax notoginseng, but also representative components which are considered to be responsible for the botanical drug’s activity (Peng et al., 2018; National Pharmacopeia Commission, 2020). As an important part of Chinese pharmaceutical standards, the Chinese Pharmacopoeia records two saponins extracted from Panax notoginseng, namely Panax Notoginseng Saponins (PNS) and Panaxatriol Saponin (PTS), where PNS is the total saponins made from the main root or rhizome of Panax notoginseng and PTS is a processed extract of the dried root and rhizome of Panax notoginseng (National Pharmacopeia Commission, 2020). PNS has anti-platelet, anti-coagulant and inhibits platelet aggregation and thrombosis (Wang T et al., 2016; Shen et al., 2017; Liu et al., 2021). PTS has anti-platelet (Qi et al., 2016; Xu et al., 2021), pro-angiogenic and cerebral perfusion enhancing effects (Hui et al., 2017). Panax notoginseng preparations (PNP) containing PNS include Xuesaitong tablet, Xuesaitong soft capsule, Xuesaitong injection and Xueshuantong injection, while PNP containing PTS include Sanqi Tongshu capsule. As another pharmaceutical form of Panax notoginseng, PNP containing the two saponins (PNS or PTS) mentioned above has been widely used in China for the treatment of coronary heart disease (CHD) and ischemic stroke (IS) (He et al., 2011; Long et al., 2020; Lyu et al., 2020).

Arterial thrombosis is the result of clot formation during atherosclerotic plaque rupture and is the predominant pathological process in CHD and IS (Koupenova et al., 2017). Increased platelet aggregation is thought to be an important pathogenesis of cardiovascular diseases such as angina pectoris and IS and is key to thrombosis (Wu and Hoak, 1975; Rauch et al., 2001). In the secondary prevention of atherosclerotic cardiovascular disease, antiplatelet therapy is an integral part of reducing ischemic events. Antiplatelet therapy is effective in reducing the incidence of adverse vascular events, but at the cost of increased risk of bleeding (Tomaniak et al., 2019; Ha et al., 2021; Pechlivani et al., 2021). The most commonly used antiplatelet agent is aspirin (Mason et al., 2005). The interaction between PNP and aspirin (ASA) is an important clinical concern. However, evidence-based medical evidence on the effects of the combination of PNP and ASA on platelet aggregation and coagulation is as of yet lacking.

The present meta-analysis comprehensively collected studies of PNP adjuvant therapy for CHD and IS, focusing on whether PNP combined with ASA produces stronger antiplatelet and anticoagulant efficacy and whether the combination increases the risk of bleeding, thus providing evidence for the safe and rational clinical use of PNP for CHD and IS.

## 2 Methods

Our meta-analysis was conducted according to the Preferred Reporting Items for Systematic Reviews and Meta-Analyses (Page et al., 2021). We registered the protocol with the International Prospective Register of Systematic Reviews under the registration number: CRD42022339234.

### 2.1 Search strategy

A comprehensive search of eight databases including CNKI, Wanfang, VIP, CBM, PubMed, Embase, Web of Science, and the Cochrane library was conducted from inception to 1 June 2022, with no language restrictions. References for relevant reviews and meta-analyses were checked to track relevant studies that might be eligible. PubMed search strategy is displayed in Table 1.

**TABLE 1:** PubMed search strategy.

Search	Query
#1	Search: (“Panax notoginseng” [Mesh]) OR (Panax notoginseng [Title/Abstract]) OR (Panax notoginsengs [Title/Abstract]) OR (notoginsengs, Panax [Title/Abstract]) OR (Panax notoginseng saponin [Title/Abstract]) OR (Panaxatriol saponin [Title/Abstract]) OR (Panax notoginseng preparation [Title/Abstract]) OR (Xueshuantong [Title/Abstract]) OR (Xuesaitong [Title/Abstract]) OR (Sanqi Tongshu [Title/Abstract])
#2	Search: (“Aspirin” [Mesh]) OR (Aspirin [Title/Abstract]) OR (Acetylsalicylic Acid [Title/Abstract]) OR (Acid, Acetylsalicylic [Title/Abstract]) OR (2-(Acetyloxy)benzoic Acid [Title/Abstract]) OR (Acylpyrin [Title/Abstract]) OR (Aloxiprimum [Title/Abstract]) OR (Colfarit [Title/Abstract]) OR (Dispril [Title/Abstract]) OR (Easprin [Title/Abstract]) OR (Ecotrin [Title/Abstract]) OR (Endosprin [Title/Abstract]) OR (Magnecyl [Title/Abstract]) OR (Micristin [Title/Abstract]) OR (Polopirin [Title/Abstract]) OR (Polopiryna [Title/Abstract]) OR (Solprin [Title/Abstract]) OR (Solupsan [Title/Abstract]) OR (Zorprin [Title/Abstract]) OR (Acetysal [Title/Abstract])
#3	Search: (“Coronary Disease” [Mesh]) OR (Coronary Disease [Title/Abstract]) OR (Coronary Diseases [Title/Abstract]) OR (Disease, Coronary [Title/Abstract]) OR (Diseases, Coronary [Title/Abstract]) OR (Coronary Heart Disease [Title/Abstract]) OR (Coronary Heart Diseases [Title/Abstract]) OR (Disease, Coronary Heart [Title/Abstract]) OR (Diseases, Coronary Heart [Title/Abstract]) OR (Heart Disease, Coronary [Title/Abstract]) OR (Heart Diseases, Coronary [Title/Abstract]) OR (ischemic heart disease [Title/Abstract])
#4	Search: (“Ischemic Stroke” [Mesh]) OR (Ischemic Stroke [Title/Abstract]) OR (Ischemic Strokes [Title/Abstract]) OR (Stroke, Ischemic [Title/Abstract]) OR (Ischaemic Stroke [Title/Abstract]) OR (Ischaemic Strokes [Title/Abstract]) OR (Stroke, Ischaemic [Title/Abstract]) OR (Cryptogenic Ischemic Stroke [Title/Abstract]) OR (Cryptogenic Ischemic Strokes [Title/Abstract]) OR (Ischemic Stroke, Cryptogenic [Title/Abstract]) OR (Stroke, Cryptogenic Ischemic [Title/Abstract]) OR (Cryptogenic Stroke [Title/Abstract]) OR (Cryptogenic Strokes [Title/Abstract]) OR (Stroke, Cryptogenic [Title/Abstract]) OR (Cryptogenic Embolism Stroke [Title/Abstract]) OR (Cryptogenic Embolism Strokes [Title/Abstract]) OR (Embolism Stroke, Cryptogenic [Title/Abstract]) OR (Stroke, Cryptogenic Embolism [Title/Abstract]) OR (Wake-up Stroke [Title/Abstract]) OR (Stroke, Wake-up [Title/Abstract]) OR (Wake up Stroke [Title/Abstract]) OR (Wake-up Strokes [Title/Abstract]) OR (Acute Ischemic Stroke [Title/Abstract]) OR (Acute Ischemic Strokes [Title/Abstract]) OR (Ischemic Stroke, Acute [Title/Abstract]) OR (Stroke, Acute Ischemic [Title/Abstract])
#5	Search: (“Platelet Aggregation” [Mesh]) OR (Platelet aggregation [Title/Abstract]) OR (Aggregation, Platelet [Title/Abstract])
#6	Search: (“Platelet Count” [Mesh]) OR (Platelet Count [Title/Abstract]) OR (Count, Platelet [Title/Abstract]) OR (Counts, Platelet [Title/Abstract]) OR (Platelet Counts [Title/Abstract]) OR (Platelet Number [Title/Abstract]) OR (Number, Platelet [Title/Abstract]) OR (Numbers, Platelet [Title/Abstract]) OR (Platelet Numbers [Title/Abstract]) OR (Blood Platelet Number [Title/Abstract]) OR (Blood Platelet Numbers [Title/Abstract]) OR (Number, Blood Platelet [Title/Abstract]) OR (Numbers, Blood Platelet [Title/Abstract]) OR (Platelet Number, Blood [Title/Abstract]) OR (Platelet Numbers, Blood [Title/Abstract]) OR (Blood Platelet Count [Title/Abstract]) OR (Blood Platelet Counts [Title/Abstract]) OR (Count, Blood Platelet [Title/Abstract]) OR (Counts, Blood Platelet [Title/Abstract]) OR (Platelet Count, Blood [Title/Abstract]) OR (Platelet Counts, Blood [Title/Abstract])
#7	Search: (“Blood Coagulation Tests” [Mesh]) OR (blood coagulation tests [Title/Abstract]) OR (Coagulation Tests Blood [Title/Abstract]) OR (Blood Coagulation Test [Title/Abstract]) OR (Coagulation Test, Blood [Title/Abstract]) OR (Test, Blood Coagulation [Title/Abstract]) OR (Tests, Blood Coagulation [Title/Abstract])
#8	Search: (“Prothrombin Time” [Mesh]) OR (prothrombin time [Title/Abstract]) OR (Prothrombin Times [Title/Abstract]) OR (Time, Prothrombin [Title/Abstract]) OR (Times, Prothrombin [Title/Abstract]) OR (Russell’s Viper Venom Time [Title/Abstract]) OR (Russell Viper Venom Time [Title/Abstract]) OR (Russells Viper Venom Time [Title/Abstract]) OR (Thrombotest [Title/Abstract]) OR (Quick Test [Title/Abstract]) OR (Test, Quick [Title/Abstract])
#9	Search: (“International Normalized Ratio” [Mesh]) OR (International Normalized Ratio [Title/Abstract]) OR (International Normalized Ratios [Title/Abstract]) OR (Normalized Ratio, International [Title/Abstract]) OR (Normalized Ratios, International [Title/Abstract]) OR (Ratio, International Normalized [Title/Abstract]) OR (Ratios, International Normalized [Title/Abstract]) OR (INR [Title/Abstract]) OR (prothrombin time international normalized ratio [Title/Abstract])
#10	Search: (“Fibrinogen” [Mesh]) OR (Fibrinogen [Title/Abstract]) OR (Blood Coagulation Factor I [Title/Abstract]) OR (Coagulation Factor I [Title/Abstract]) OR (Factor I, Coagulation [Title/Abstract]) OR (Factor I [Title/Abstract]) OR (gamma-Fibrinogen [Title/Abstract]) OR (gamma Fibrinogen [Title/Abstract])
#11	Search: (“fibrin fragment D” [Supplementary Concept]) OR (fibrin fragment D [Title/Abstract]) OR (D-dimer fibrin [Title/Abstract]) OR (D-dimer fragments [Title/Abstract]) OR (fibrin fragment D1 dimer [Title/Abstract]) OR (fibrin fragment DD [Title/Abstract]) OR (D-dimer [Title/Abstract]) OR (fibrin fragment D-dimer [Title/Abstract])
#12	Search: (randomized controlled trial [Publication Type] OR randomized [Title/Abstract] OR random [Title/Abstract])
#13	Search: #3 OR #4
#14	Search: #5 OR #6 OR #7 OR #8 OR #9 OR #10 OR #11
#15	Search: #1 AND #2 AND #12 AND #13 AND #14

### 2.2 Eligibility criteria

We selected these studies based on the following inclusion criteria: 1) randomized controlled trials (RCTs); 2) patients with CHD and IS; 3) antiplatelet regimen of PNP plus ASA in the intervention group and ASA alone in the control group; 4) studies reporting one or more of six outcomes: platelet aggregation rate (PAgR), platelet count (PLT), prothrombin time (PT), prothrombin time-based international normalized ratio (PT-INR), fibrinogen (FIB), and D-dimer (DD).

Exclusion criteria: 1) required data were not obtained; 2) no outcome measures of interest; 3) no full text or conference abstracts; 4) duplicate publications or a second publication of the same trial.

### 2.3 Study selection

Two reviewers (LD and YZ) independently reviewed the titles and abstracts of the studies according to the eligibility criteria. If potential studies met the criteria, further full-text evaluation was required. Studies that remained controversial would be arbitrated by a third researcher (YJ). We used NoteExpress software (Version 3.2) to manage the retrieved records.

### 2.4 Data extraction

Two reviewers (LD and YZ) extracted the following data independently of each other: first author, year of publication, characteristics of patients (sample, proportion of male and mean age), disease, antiplatelet regimen, primary outcome (PAgR, PLT, PT, PT-INR, FIB, and DD) and secondary outcome (upper gastrointestinal bleeding, bulbar conjunctival hemorrhage, hematuria, subcutaneous hemorrhage, positive fecal occult blood, gastrointestinal side effects, urticaria), and then cross-checked the extracted data.

### 2.5 Risk of bias of studies

Two reviewers (LD and YZ) independently assessed the risk of bias of the studies according to the Cochrane risk-of-bias tool (ROB 2) (Sterne et al., 2019). We judged the studies to be at low risk of bias, with some concerns or high risk of bias, and any disagreements should be adjudicated by a third reviewer (YJ).

### 2.6 Statistical analysis

We adopted Review Manager (Version 5.4, The Cochrane Collaboration, Copenhagen, 2020), R software (Version 4.0.1; R Foundation for Statistical Computing, Vienna, Austria), and Stata/SE (Version 15.1; StataCorp, College Station, Texas, United States) to analyze data and generate figures. If heterogeneity was low (*p* values ≥0.1 and I^2^ < 50%), a fixed-effects model was adopted, otherwise a random-effects model was adopted. For continuous and dichotomous data, we calculated their weighted mean difference (WMD) and risk ratio (RR), respectively. Considering the possible differences in the effects of the two saponins (PNS and PTS), we divided PNP into two categories according to the saponin category and performed subgroup analysis. Sensitivity analysis was performed using the leave-one-out approach to test the stability and reliability of the findings. When more than ten studies reported the outcome, the presence of publication bias was detected by funnel plot, Egger’ test and Begg’ test.

## 3 Results

### 3.1 Study selection and identification

**FIGURE 1:**
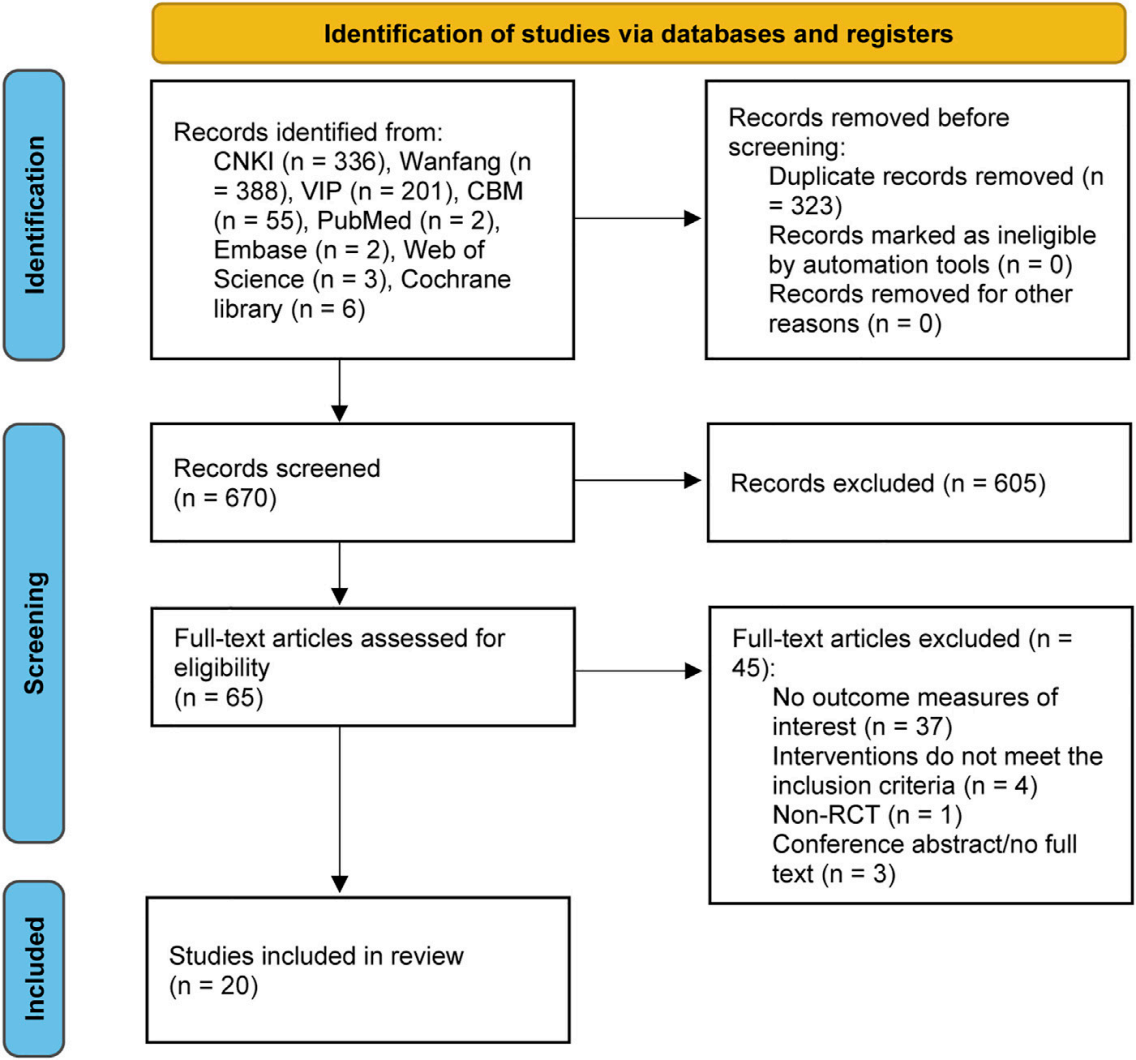
PRISMA flow diagram of literature selection and identification. *PRISMA,* Preferred reporting items for systematic reviews and meta-analysis.

A total of 993 records were identified from the eight databases. After removing 323 duplicate records, the titles and abstracts of 670 records were screened, and then 605 records were excluded. We evaluated the full text of 65 potentially eligible studies. Finally, 20 (Zhou et al., 2008; Zong et al., 2009; Zhang et al., 2010; Li, 2011; Zhong et al., 2011; Piao, 2012; Zhang, 2013; Cheng et al., 2014; Yang, 2014; Yu et al., 2014; Yan and Cui, 2015; Hu, 2016; Wu, 2016; Ma et al., 2017; Shen, 2017; Chen et al., 2018; Xue, 2018; Sun, 2019; Li, 2021; Wang et al., 2021) studies were identified. Figure 1 shows the detailed screening process.

### 3.2 Study characteristics

The characteristics of the 20 studies are detailed in Table 2. The year of publication of the 20 studies ranged from 2008 to 2021. A total of 2216 patients were included in the 20 studies, 1110 in the PNP plus ASA group and 1106 in the ASA group, all of whom were middle-aged and elderly. In 15 studies (Zong et al., 2009; Zhang et al., 2010; Zhong et al., 2011; Zhang, 2013; Cheng et al., 2014; Yang, 2014; Yu et al., 2014; Hu, 2016; Wu, 2016; Ma et al., 2017; Chen et al., 2018; Xue, 2018; Sun, 2019; Li, 2021; Wang et al., 2021), the component of PNP was PNS, whereas in 5 studies (Zhou et al., 2008; Li, 2011; Piao, 2012; Yan and Cui, 2015; Shen, 2017), the component of PNP was PTS.

**TABLE 2:** Characteristic of selected studies.

	Sample	Male (%)	Mean age (years)	Disease	Component of PNP	Antiplatelet treatment regimen				Outcome	
Study	PNP plus ASA/ASA	PNP plus ASA/ASA	PNP plus ASA/ASA			PNP plus ASA	ASA	Intervention time	Test time	Primary	Secondary
Zhou et al. (2008)	40/40	52.5/55	69.90 ± 10.71/71.16 ± 10.60	ischemic stroke	PTS	Sanqi Tongshu capsule 200 mg tid + ASA 100 mg qd	ASA 100 mg qd	6 m	3m; 6 m	PAgR	①; ②; ③; ④; ⑤
Li, (2011)	27/28	48.15/53.57	74.25 ± 6.32/76.32 ± 9.04	coronary heart disease	PTS	Sanqi Tongshu capsule 200 mg tid + ASA 100 mg qd	ASA 100 mg qd	6 m	6 m	PAgR; PT; PT-INR; DD	④; ⑥; ⑦
Yan and Cui, (2015)	28/29	NR/NR	NR/NR	ischemic stroke	PTS	Sanqi Tongshu capsule 200 mg tid + ASA 100 mg qd	ASA 100 mg qd	6 m	6 m	PAgR; PT; PT-INR; DD	⑦
Wu, (2016)	100/100	55/57	58.64 ± 7.13/58.58 ± 7.08	ischemic stroke	PNS	Xuesaitong injection 400 mg qd + ASA 100 mg qd	ASA 100 mg qd	2w	2w	PAgR; PLT; FIB; DD	⑤
Zong et al. (2009)	40/40	67.5/62.5	51 ± 11/53 ± 9	angina pectoris	PNS	Xuesaitong injection 400 mg qd + ASA 50 mg qd	ASA 50 mg qd	2w	2w	PAgR	NR
Cheng et al. (2014)	33/30	54.55/56.67	59.8 ± 6.7/60.6 ± 6.4	acute ischemic stroke	PNS	Xuesaitong injection 400 mg qd + ASA 100 mg qd	ASA 100 mg qd	2w	2w	PAgR; PLT; FIB; DD	NR
Li, (2021)	45/40	64.44/65	59 ± 12.59/58 ± 8.15	acute ischemic stroke	PNS	Xuesaitong soft capsule 660 mg bid + ASA 100 mg qd	ASA 100 mg qd	3 m	3 m	PLT; PT	NR
Chen et al. (2018)	60/60	51.67/50	63.87 ± 11.02/64.29 ± 11.36	ischemic stroke	PNS	Xuesaitong tablet 50–100 mg tid + ASA 100–200 mg qd	ASA 100–200 mg qd	3w	3w	PAgR	NR
Zhang (2013)	40/40	52.5/55	55.14 ± 6.28/55.36 ± 6.19	unstable angina	PNS	Xuesaitong injection 400 mg qd + ASA 100 mg qd	ASA 100 mg qd	2w	2w	PAgR; FIB	NR
Piao (2012)	28/29	53.57/55.17	72.89 ± 8.45/75.62 ± 6.30	ischemic stroke	PTS	Sanqi Tongshu capsule 200 mg tid + ASA 100 mg qd	ASA 100 mg qd	6 m	6 m	PAgR; PT; PT-INR; DD	④; ⑥; ⑦
Yang (2014)	50/50	58/54	65.55 ± 12.72/64.52 ± 12.36	ischemic stroke	PNS	Xuesaitong soft capsule 660 mg bid + ASA 100–300 mg qd	ASA 100–300 mg qd	3w	3w	PLT	NR
Sun (2019)	168/168	57.14/53.57	64.2 ± 8.7/65.4 ± 8.4	ischemic stroke	PNS	Xuesaitong injection 400 mg qd + ASA 100 mg qd	ASA 100 mg qd	2w	2w	PLT	NR
Wang et al. (2021)	20/20	85/90	57.5 ± 4.07/55.75 ± 5.14	stable coronary heart disease	PNS	Xuesaitong soft capsule 660 mg bid + ASA 100 mg qd	ASA 100 mg qd	2 m	2 m	PAgR	NR
Xue (2018)	36/36	52.78/55.56	58.18 ± 6.52/57.89 ± 6.85	ischemic stroke with middle cerebral artery stenosis	PNS	Xuesaitong injection 400 mg qd + ASA 100 mg qd	ASA 100 mg qd	4w	4w	FIB	④
Shen (2017)	123/123	51.22/50.41	64.33 ± 10.25/64.35 ± 10.64	ischemic stroke	PTS	Sanqi Tongshu capsule 200 mg tid + ASA 100–300 mg qd	ASA 100–300 mg qd	3w	3w	PAgR	NR
Ma et al. (2017)	40/40	65/50	55.82 ± 12.11/57.23 ± 10.21	acute ischemic stroke	PNS	Xueshuantong injection 500 mg qd + ASA 100 mg qd	ASA 100 mg qd	2w	2w	FIB	NR
Hu (2016)	40/40	55/52.5	61.2 ± 12.3/60.1 ± 12.6	acute ischemic stroke	PNS	Xueshuantong injection 400 mg qd + ASA 100 mg qd	ASA 100 mg qd	2w	2w	FIB; DD	NR
Yu et al. (2014)	34/34	52.94/58.82	58.5 ± 9.7/55.1 ± 11.2	acute ischemic stroke	PNS	Xueshuantong injection 500 mg qd + ASA 100 mg qd	ASA 100 mg qd	2w	2w	FIB	NR
Zhong et al. (2011)	38/38	56.58 (all)	NR/NR	ischemic stroke	PNS	Xuesaitong tablet 50–100 mg tid + ASA 100 mg qd	ASA 100 mg qd	12 m	12 m	PAgR	NR
Zhang et al. (2010)	120/121	56.67/53.72	67.2/65.3	acute ischemic stroke	PNS	Xuesaitong tablet 200 mg tid + ASA 100 mg qd	ASA 100 mg qd	12 m	12 m	PAgR	NR

Note: ① upper gastrointestinal bleeding; ② bulbar conjunctival hemorrhage; ③ hematuria; ④ gastrointestinal side effects; ⑤ urticaria; ⑥ subcutaneous hemorrhage; ⑦ positive fecal occult blood.

Abbreviations: PNP, panax notoginseng preparation; ASA, aspirin; NR, not reported; PTS, panaxatriol saponin; PNS, panax notoginseng saponin; tid, three times a day; qd, once daily; bid, twice a day; PAgR, platelet aggregation rate; PLT, platelet count; PT, prothrombin time; PT-INR, prothrombin time-based international normalized ratio; FIB, fibrinogen; DD, D-dimer.

### 3.3 Risk of bias assessment

**FIGURE 2:**
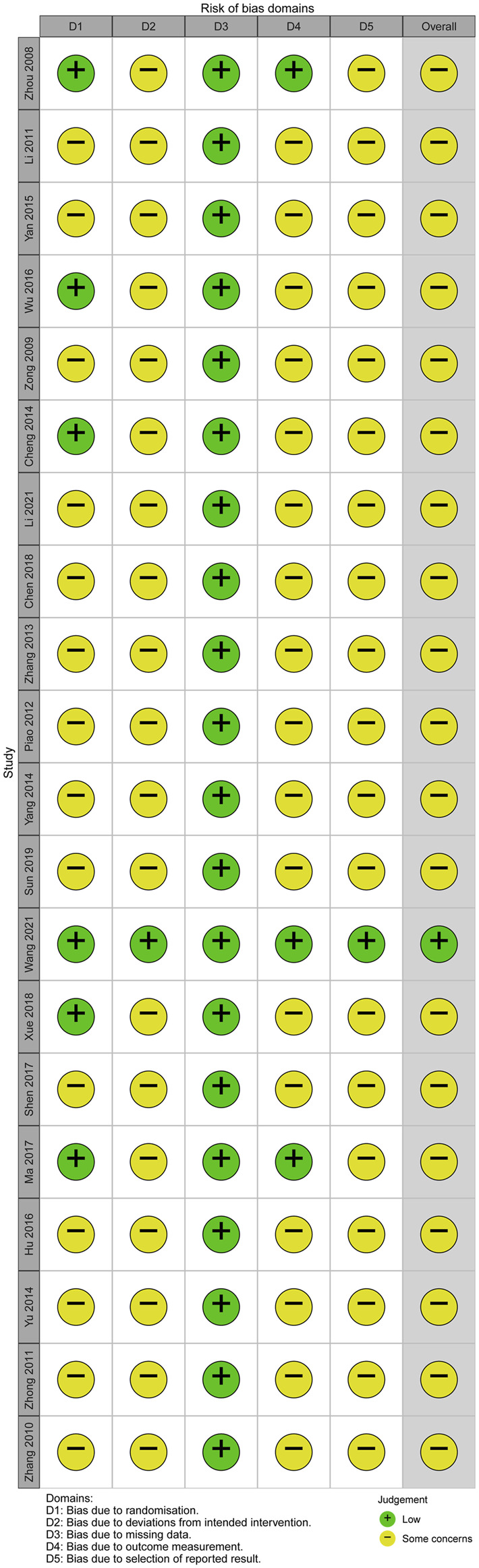
Risk of bias graph.

**FIGURE 3:**
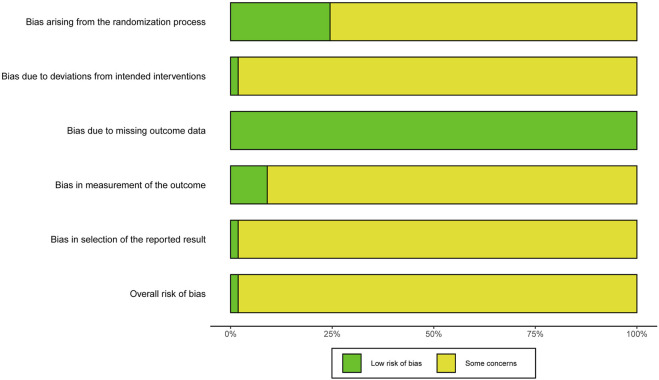
Risk of bias summary.

The results of the risk of bias assessment are presented in Figures 2, 3. Six RCTs (Zhou et al., 2008; Cheng et al., 2014; Wu, 2016; Ma et al., 2017; Xue, 2018; Wang et al., 2021) showed low risk on randomization process. One RCT (Wang et al., 2021) showed low risk on deviations from intended intervention. All RCTs showed low risk on missing data. Two RCTs (Ma et al., 2017; Wang et al., 2021) showed low risk on outcome measurement. Only one RCT (Wang et al., 2021) showed low risk on selection of reported result.

### 3.4 Meta-analysis of PAgR and PLT

**FIGURE 4:**
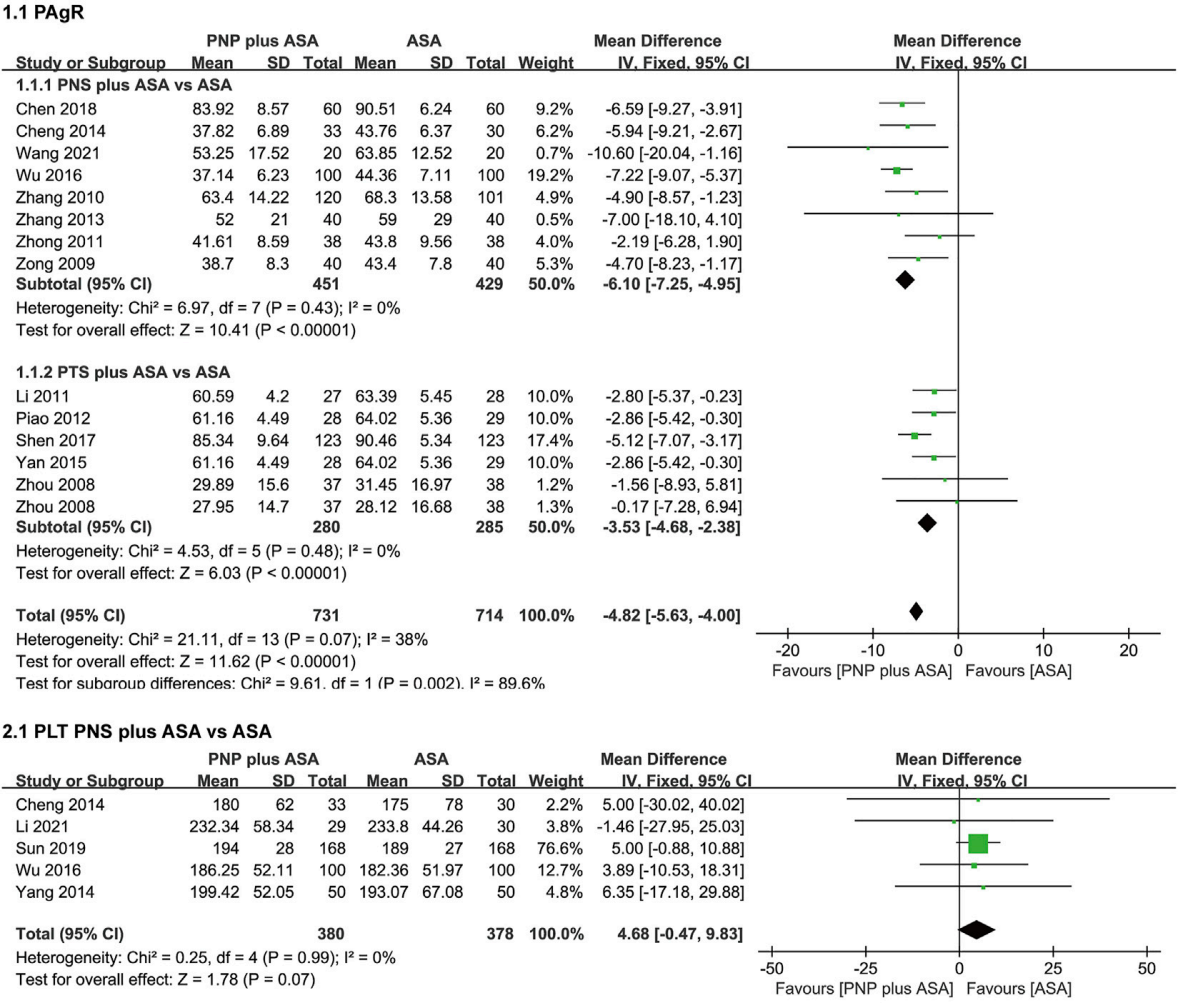
Forest plots of PAgR and PLT. *PAgR,* platelet aggregation rate; *PLT,* platelet count.

The meta-analysis results of PAgR and PLT are shown in Figure 4.

Thirteen studies (Zhou et al., 2008; Zong et al., 2009; Zhang et al., 2010; Li, 2011; Zhong et al., 2011; Piao, 2012; Zhang, 2013; Cheng et al., 2014; Yan and Cui, 2015; Wu, 2016; Shen, 2017; Chen et al., 2018; Wang et al., 2021) included 14 sets of data comparing PAgR in the PNP plus ASA group and the ASA group. There was moderate heterogeneity in the 14 sets of data (*p* = 0.07, I^2^ = 38%), so we performed a subgroup analysis according to saponin category. After subgroup analysis, the moderate heterogeneity of PAgR was eliminated. The results of the subgroup analysis were as follows, and eight studies (Zong et al., 2009; Zhang et al., 2010; Zhong et al., 2011; Zhang, 2013; Cheng et al., 2014; Wu, 2016; Chen et al., 2018; Wang et al., 2021) compared the PAgR of the PNS plus ASA group with that of the ASA group. The PAgR of the PNS plus ASA group was lower than that of the ASA group [WMD = −6.10 (−7.25, -4.95), *p* < 0.00001], with no heterogeneity among the eight studies (*p* = 0.43, I^2^ = 0%). Five studies (Zhou et al., 2008; Li, 2011; Piao, 2012; Yan and Cui, 2015; Shen, 2017) included six sets of data comparing PAgR in the PTS plus ASA group and the ASA group. PAgR of the PTS plus ASA group was lower than that of the ASA group [WMD = −3.53 (−4.68, −2.38), *p* < 0.00001], with no heterogeneity among the six sets of data (*p* = 0.48, I^2^ = 0%).

Five studies (Cheng et al., 2014; Yang, 2014; Wu, 2016; Sun, 2019; Li, 2021) compared PLT between the PNS plus ASA group and the ASA group. There was no significant difference in PLT between the PNS plus ASA group and the ASA group [WMD = 4.68 (−0.47, 9.83), *p* = 0.07], and no heterogeneity among the five studies (*p* = 0.99, I^2^ = 0%).

### 3.5 Meta-analysis of PT, PT-INR, FIB and DD

**FIGURE 5:**
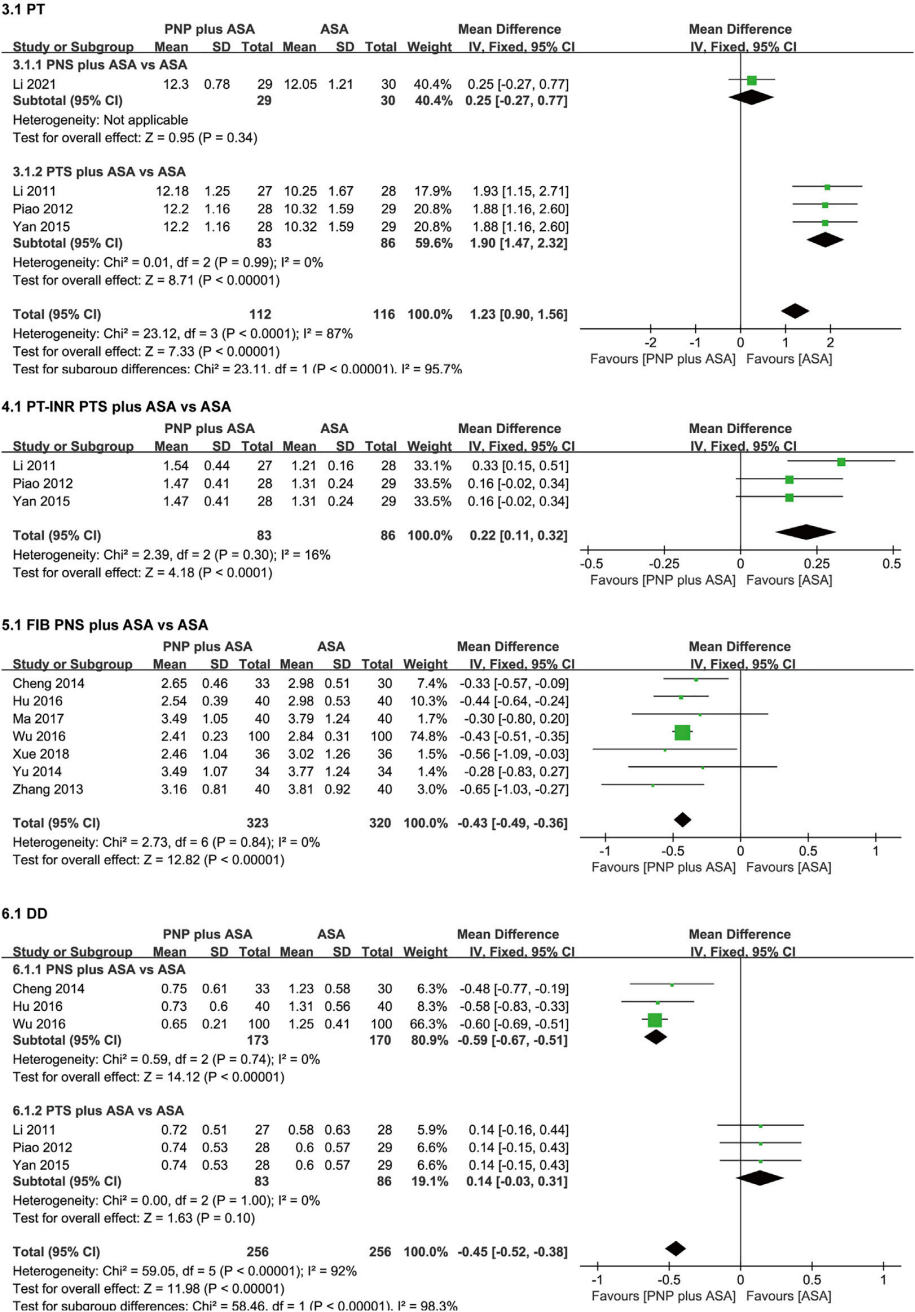
Forest plots of PT, PT-INR, FIB, and DD. *PT,* prothrombin time; *PT-INR,* prothrombin time-based international normalized ratio; *FIB,* fibrinogen; *DD,* D-dimer.

The meta-analysis results of PT, PT-INR, FIB, and DD are shown in Figure 5.

Four studies (Li, 2011; Piao, 2012; Yan and Cui, 2015; Li, 2021) compared PT in the PNP plus ASA group and the ASA group. Considerable heterogeneity existed in the four studies (*p* < 0.0001, I^2^ = 87%), so we performed a subgroup analysis according to saponin category. After subgroup analysis, considerable heterogeneity in PT was eliminated. Subgroup analysis were as follows, one study (Li, 2021) compared PT between the PNS plus ASA group and the ASA group. There was no significant difference in PT between the PNS plus ASA group and the ASA group [WMD = 0.25 (-0.27, 0.77), *p* = 0.34]. Three studies (Li, 2011; Piao, 2012; Yan and Cui, 2015) compared PT between the PTS plus ASA group and the ASA group. PT in the PTS plus ASA group was higher than that in the ASA group [WMD = 1.90 (1.47, 2.32), *p* < 0.00001], with no heterogeneity among the three studies (*p* = 0.99, I^2^ = 0%).

Three studies (Li, 2011; Piao, 2012; Yan and Cui, 2015) compared PT-INR between the PTS plus ASA group and the ASA group. PT-INR in the PTS plus ASA group was higher than that in the ASA group [WMD = 0.22 (0.11, 0.32), *p* < 0.0001], with no heterogeneity among the three studies (*p* = 0.30, I^2^ = 16%).

Seven studies (Zhang, 2013; Cheng et al., 2014; Yu et al., 2014; Hu, 2016; Wu, 2016; Ma et al., 2017; Xue, 2018) compared FIB between the PNS plus ASA group and the ASA group. FIB in the PNS plus ASA group was lower than that in the ASA group [WMD = −0.43 (−0.49, −0.36), *p* < 0.00001], with no heterogeneity among the seven studies (*p* = 0.84, I^2^ = 0%).

Six studies (Li, 2011; Piao, 2012; Cheng et al., 2014; Yan and Cui, 2015; Hu, 2016; Wu, 2016) compared DD in the PNP plus ASA group and the ASA group. There was considerable heterogeneity in the six studies (*p* < 0.00001, I^2^ = 92%), so we performed a saponin category-based subgroup analysis. After subgroup analysis, considerable heterogeneity in DD was eliminated. Subgroup analysis were as follows, three studies (Cheng et al., 2014; Hu, 2016; Wu, 2016) compared DD in the PNS plus ASA group and the ASA group. DD in the PNS plus ASA group was lower than that in the ASA group [WMD = −0.59 (−0.67, −0.51), *p* < 0.00001], and there was no heterogeneity in the three studies (*p* = 0.74, I^2^ = 0%). Three studies (Li, 2011; Piao, 2012; Yan and Cui, 2015) compared DD between the PTS plus ASA group and the ASA group. There was no significant difference in DD between the PTS plus ASA group and the ASA group [WMD = 0.14 (−0.03, 0.31), *p* = 0.1], and there was no heterogeneity among the three studies (*p* = 1, I^2^ = 0%).

### 3.6 Meta-analysis of adverse reactions

**FIGURE 6:**
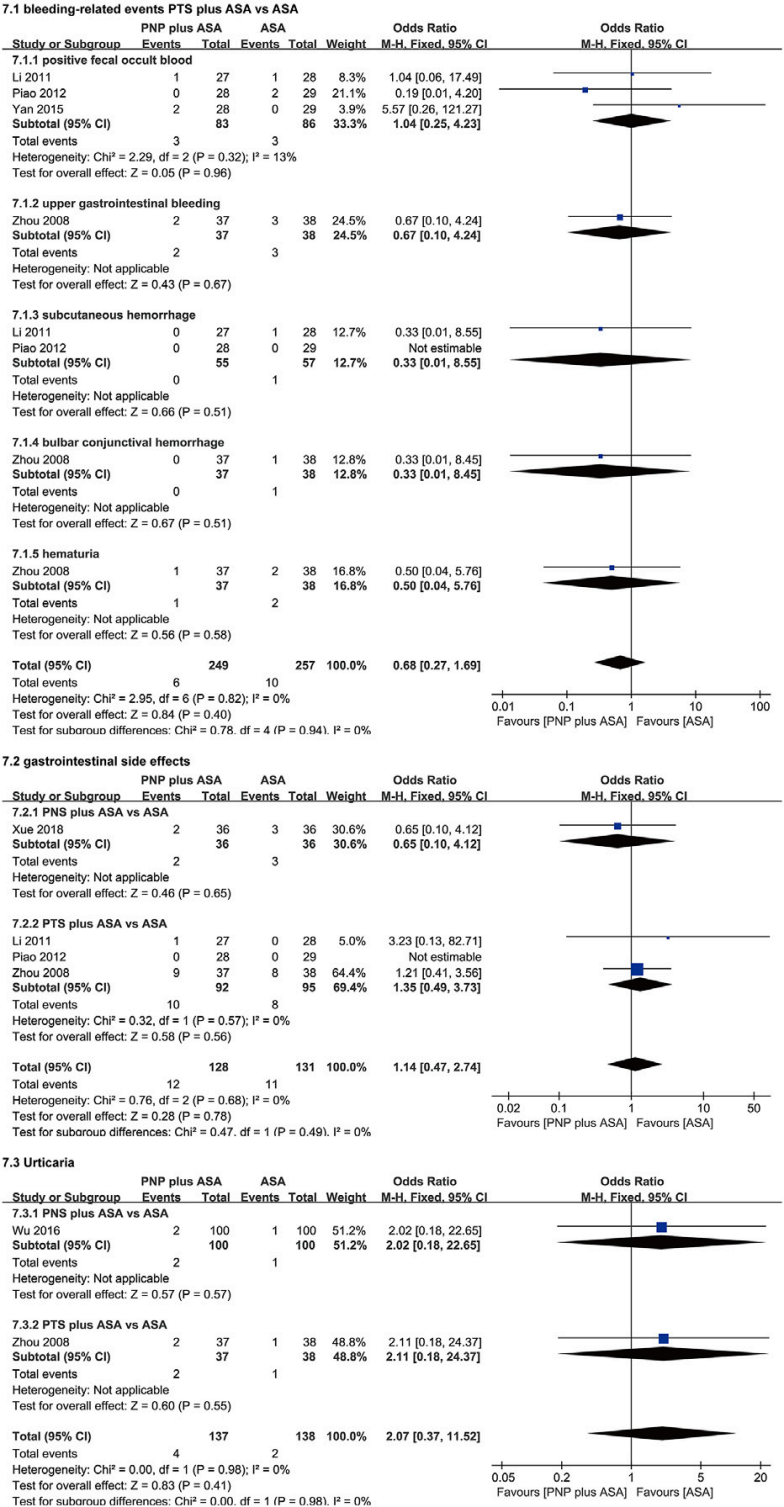
Forest plots of adverse reactions.

The meta-analysis results of adverse reactions are shown in Figure 6; Table 3.

**TABLE 3:** Meta-analysis results of adverse reactions.

Adverse reaction	Comparision	Number of studies	Effect estimate	Heterogeneity	Test for overall effect
Positive fecal occult blood	PTS plus ASA vs. ASA	3	1.04 [0.25, 4.23]	*p* = 0.35; I^2^ = 10%	Z = 0.05; *p* = 0.96
Upper gastrointestinal bleeding	PTS plus ASA vs. ASA	1	0.67 [0.10, 4.24]	Not applicable	Z = 0.43; *p* = 0.67
Subcutaneous hemorrhage	PTS plus ASA vs. ASA	2	0.33 [0.01, 8.55]	Not applicable	Z = 0.66; *p* = 0.51
Bulbar conjunctival hemorrhage	PTS plus ASA vs. ASA	1	0.33 [0.01, 8.45]	Not applicable	Z = 0.67; *p* = 0.51
Hematuria	PTS plus ASA vs. ASA	1	0.50 [0.04, 5.76]	Not applicable	Z = 0.56; *p* = 0.58
Gastrointestinal side effects	PNS plus ASA vs. ASA	1	0.65 [0.10, 4.12]	Not applicable	Z = 0.46; *p* = 0.65
	PTS plus ASA vs. ASA	3	1.35 [0.49, 3.73]	*p* = 0.57; I^2^ = 0%	Z = 0.58; *p* = 0.56
Urticaria	PNS plus ASA vs. ASA	1	2.02 [0.18, 22.65]	Not applicable	Z = 0.57; *p* = 0.57
	PTS plus ASA vs. ASA	1	2.11 [0.18, 24.37]	Not applicable	Z = 0.60; *p* = 0.55

Abbreviations: PTS, panaxatriol saponin; ASA, aspirin; PNS, panax notoginseng saponin.

There were no significant differences between the PTS plus ASA group and the ASA group in terms of bleeding-related events [positive fecal occult blood (*p* = 0.96); upper gastrointestinal bleeding (*p* = 0.67); subcutaneous hemorrhage (*p* = 0.51); bulbar conjunctival hemorrhage (*p* = 0.51); hematuria (*p* = 0.58)]. There were no significant differences between the PNP plus ASA group and the ASA group in terms of gastrointestinal side effects (PNS, *p* = 0.65; PTS, *p* = 0.56) and urticaria (PNS, *p* = 0.57; PTS, *p* = 0.55).

### 3.7 Sensitivity analysis and publication bias

Although moderate or considerable heterogeneity between studies reporting PAgR, PT, and DD was eliminated after subgroup analysis, we still performed sensitivity analysis using the leave-one-out approach to verify the stability and reliability of the findings and found that all pooled results were robust.

**FIGURE 7:**
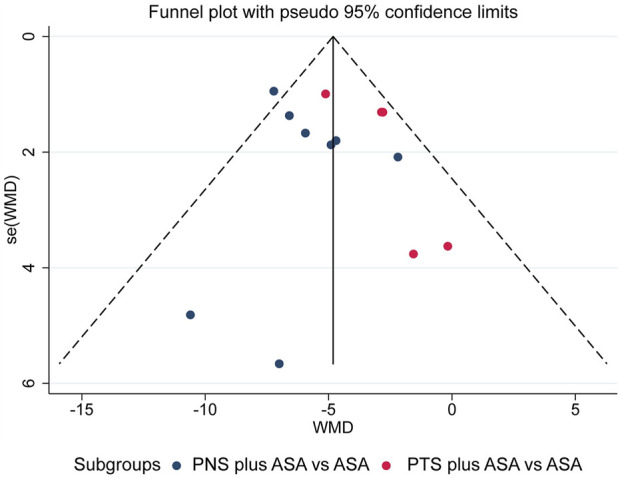
Funnel plot of comparison of PAgR between PNS plus ASA group or PTS plus ASA group and ASA group. *PAgR,* platelet aggregation rate; *PNS,* Panax Notoginseng Saponins; *ASA,* aspirin; *PTS,* Panaxatriol Saponin.

Thirteen studies (Zhou et al., 2008; Zong et al., 2009; Zhang et al., 2010; Li, 2011; Zhong et al., 2011; Piao, 2012; Zhang, 2013; Cheng et al., 2014; Yan and Cui, 2015; Wu, 2016; Shen, 2017; Chen et al., 2018; Wang et al., 2021) included 14 sets of data comparing the PAgR of PNP plus ASA and ASA alone, so we tested for publication bias and found no evidence of publication bias according to funnel plot, Egger’ test and Begg’ test (Figure 7, Egger’s test, *p* = 0.462; Begger’s test, *p* = 1.000). Studies reporting other outcomes were not adequate, so we were unable to perform the test.

## 4 Discussion

### 4.1 Summary of findings

The meta-analysis included 20 studies with 2216 patients. To sum up, our analysis showed that PNP (PNS or PTS) plus ASA had a significantly stronger inhibitory effect on PAgR than ASA alone; PNS plus ASA was better than ASA alone in reducing FIB and DD, while there was no significant difference in PLT and PT; PTS plus ASA was better than ASA alone in prolonging PT and PT-INR, while there was no significant difference in DD; in terms of adverse reactions, there was no significant difference between PTS plus ASA and ASA alone in terms of bleeding-related events, nor was there a significant difference between PNP (PNS or PTS) plus ASA and ASA alone in terms of gastrointestinal side effect and urticaria.

### 4.2 Clinical implications

Our meta-analysis focused on the effects of the combination of PNP and ASA on platelet aggregation and coagulation and demonstrated that the combination had synergistic antiplatelet and anticoagulant efficacy. It was found that ASA increases the absorption of PNS by disrupting tight junction proteins to open intercellular space (Tian et al., 2018), while PNS also promotes the absorption of ASA in the gastrointestinal tract (Tian et al., 2017), in addition, a recent study (Wang et al., 2021) showed that the combination of PNS and ASA enhanced the antiplatelet effect of ASA *via* the AA/COX-1/TXB_2_ pathway, which may partially explain the synergistic effect of the combination of PNP and ASA. Notably, there was no significant difference in bleeding-related events between the PTS plus ASA group and the ASA group. The combination of ASA with other antiplatelet or anticoagulant drugs produces a stronger antithrombosis effect, but also a higher risk of bleeding (Kozieł and Lip, 2019). Therefore, it is necessary to balance the pros and cons and to rationalize the use of antiplatelet drugs in people at high risk of bleeding. Our results suggested that the combination of PNP, of which PTS is the main component, with ASA did not increase the risk of bleeding, which may be beneficial for patients with atherothrombotic disease at high risk of bleeding, and the underlying mechanisms need to be further explored in basic research. Results of this meta-analysis also showed that the combination of PNP and ASA did not increase gastrointestinal side effects. Aspirin can cause gastric mucosal damage, and in some patients, the cardiovascular benefits of low-dose aspirin may be offset by gastrointestinal risks (Sostres and Lanas, 2011). *In vivo* studies in rats with myocardial infarction have shown that the combination of PNS and ASA can alleviate ASA-related gastrointestinal side effects *via* the AA/PG pathway. Although PNP was not found to reduce the gastrointestinal side effects of aspirin in this meta-analysis, it at least did not increase such side effects. Results of this meta-analysis may provide clinicians and medical professionals with evidence-based medical evidence when choosing PNP as an adjunct to the treatment of CHD or IS.

## 5 Strength and limitation

This is the first meta-analysis to focus on the potential interaction of PNP combined with ASA in platelet aggregation and coagulation. In addition to this, in previous meta-analyses (Shang et al., 2013; Song et al., 2017; Duan et al., 2018; Gao et al., 2019), only PNP with PNS as the main active component and not PNP with PTS as the main active component were included, but our meta-analysis included PNP with both two saponins as the main active components and subgroup analyses were performed according to the saponin category.

However, some limitations of this meta-analysis should be noted. First, most of the included literatures were in Chinese and only one was in English, and the possibility of language bias could not be excluded. Second, the methodological quality of the literatures included in the analysis were not high, which might reduce the reliability of our findings to some extent. Third, some of the outcomes of the meta-analysis involved only one of the two saponin, PTS or PNS, such as PLT, PT-INR, and FIB, especially for bleeding-related events, which indicate that the current attention to these areas is still insufficient.

## 6 Conclusion

Preliminary evidence from our meta-analysis shows that compared with ASA alone, combined with PNP might produce stronger anti-platelet aggregation and anticoagulation effects without increasing the risk of bleeding, gastrointestinal side effects, and urticaria. However, given the potential biases of included studies, the evidence may be limited and requires further evaluation. Therefore, such preliminary studies need to pay more attention to randomization, blinding and biased reporting to provide evidence of higher methodological quality.

## Data Availability

The original contributions presented in the study are included in the article/Supplementary Material, further inquiries can be directed to the corresponding authors.
